# Hepatic cavernous hemangioma decellularized extracellular matrix/GelMA composite hydrogel promotes angiogenesis via the ITGA9–FAK–ERK1/2 axis

**DOI:** 10.1016/j.mtbio.2026.102976

**Published:** 2026-02-28

**Authors:** Zongbo Dai, Xuejian Li, Meiqi Jin, Wanting Liu, Xiaohang Li, Chengshuo Zhang, Feng Li, Jianzhen Lin, Ziyi Zhou, Xiaoting Sun, Tianlin Wang, Huazhe Yang, Jialin Zhang

**Affiliations:** a Department of Hepatobiliary Surgery, The First Hospital of China Medical University, Shenyang, 110001, China; b School of Intelligent Medicine, China Medical University, Shenyang, 110121, China; c School of Forensic Medicine, China Medical University, Shenyang, 110121, China

**Keywords:** Angiogenesis, Hydrogel, Extracellular matrix, Tissue engineering, GelMA

## Abstract

Effective angiogenesis is crucial for tissue engineering and regenerative medicine. However, current strategies such as growth factor or endothelial cell (EC) delivery often face challenges in inducing stable and efficient vascularization. Inspired by mimicking matrix composition and physical architecture of vascular hyperplastic tissues, we developed a novel composite hydrogel composed of decellularized extracellular matrix from human hepatic cavernous hemangioma (HCH dECM) and gelatin methacryloyl (GelMA) to promote angiogenesis for tissue engineering applications. The HCH dECM/GelMA hydrogel exhibited improved mechanical stability, uniform porosity, and retention of pro-angiogenic basement membrane components, as confirmed by proteomic and biomechanical analyses. In vitro, the hydrogel significantly enhanced the viability, proliferation, migration, and tube formation of human umbilical vein endothelial cells (HUVECs), outperforming Matrigel in key morphological metrics. In a mouse subcutaneous implantation model, the HCH dECM/GelMA hydrogel robustly induced vascularization by recruiting host endothelial cells, as evidenced by increased CD31^+^ and α-SMA^+^ areas. Mechanistic investigations revealed that the hydrogel upregulates integrin alpha 9 (ITGA9), activating the FAK–ERK1/2 signaling pathway and enhancing the expression of angiogenic cytokines such as VEGFA. Knockdown of ITGA9 abolished these pro-angiogenic effects, confirming the essential role of the ITGA9–FAK–ERK1/2 axis. This work presents a human-derived, bioactive hydrogel with significant potential for vascular regeneration and clinical translation.

## Highlights

• A novel ECM source: human hepatic cavernous hemangioma (HCH) as a bioactive ECM reservoir enriched with pro-angiogenic components.
• The HCH dECM/GelMA hydrogel exhibits enhanced mechanical stability and robustly promotes angiogenesis both in vitro and in vivo.
• The composite hydrogel outperforms Matrigel in key metrics of endothelial tube formation.
• The pro-angiogenic effect is mediated via the ITGA9–FAK–ERK1/2 signaling axis.

## 1 Introduction

Angiogenesis is the process of blood vessel formation initiated by the sprouting and fusion of endothelial cells (ECs) [1]. The induction of angiogenesis plays a crucial role in tissue engineering and regenerative medicine [2]. For example, the microenvironment of refractory wounds is typically characterized by local ischemia and chronic hypoxia, and effective angiogenesis is essential to break this vicious cycle [3]. Vascular-rich granulation tissue provides the foundation for wound filling and re-epithelialization [4]. In the field of organoid construction and transplantation, insufficient oxygen and nutrient supply has become a bottleneck limiting organoid viability and function [5]. A well-developed vascular network not only transports nutrients and removes metabolic waste but also serves as a structural basis for rebuilding endocrine systems [6]. Furthermore, promoting angiogenesis is a fundamental therapeutic strategy for vascular dysfunction diseases such as coronary heart disease and cerebral infarction [7]. Neovascularization establishes collateral circulation, acting as a biological bypass to circumvent occluded vessels and restore blood perfusion to ischemic tissues [8].

Significant progress has been made in strategies for inducing angiogenesis. For instance, researchers genetically modify stem cells to overexpress pro-angiogenic factors like vascular endothelial growth factor A (VEGFA), or apply degradable polymer materials as cytokine carriers for transplantation into ischemic sites [9,10]. Others have cultivated three-dimensional vascular networks containing endothelial and mural cells in vitro, with the aim of integrating them into the host's microcirculation system after transplantation [11]. While these approaches have demonstrated considerable promise in promoting angiogenesis within target tissues, certain challenges remain to be addressed for their broader application. First, free angiogenic substances often lack stability in vivo, and the release kinetics of angiogenic factors from gene-edited stem cells or biomaterials frequently differ significantly from physiological conditions [12]. Second, due to factors such as immunogenicity, the viability and function of exogenous endothelial cells decline markedly after implantation, and their integration efficiency with the host's vascular system is generally low [13]. To overcome these challenges, functionalized biomimetic scaffolds that can simulate the natural extracellular environment, provide physical support, and controllably release factors have emerged as a promising solution.

The extracellular matrix (ECM) is a complex three-dimensional macromolecular network composed of collagen, elastin, proteoglycans, glycosaminoglycans, glycoproteins, and various growth factors [14]. Cellular behaviors such as survival, proliferation, migration, and differentiation are all supported by the ECM [15]. Bioactive materials derived from native ECM can more effectively replicate the physiological cellular microenvironment, thereby facilitating desired cellular functions [16]. In recent studies, hydrogels, electrospun scaffolds, and bioprinted scaffolds have become the main categories of ECM-based bioactive scaffolds [17]. ECM hydrogels are hydrophilic polymers derived from natural tissues through decellularization, digestion, and dissolution [18]. Owing to their highly biomimetic aqueous environment, excellent morphological adaptability, and tunable stiffness, ECM hydrogels have been increasingly used for culturing and transplanting target cells [19]. Previous studies suggest that ECM is most suitable for supporting the survival of corresponding cell types due to the specificity and heterogeneity of different tissues [20]. For example, injectable ECM hydrogel derived from cartilage has been applied in treating intervertebral disc degeneration, and pancreatic decellularized ECM (dECM) has been used in constructing 3D-printed islet organoids [21,22]. Recently, dECM hydrogels derived from perinatal tissues such as the placenta, umbilical cord, and amniotic membrane have been shown to promote vascular formation both in vivo and in vitro [23,24]. Certain basement membrane components in these hydrogels are thought to contribute to vascularization, though the specific molecular mechanisms remain unclear [25].

Although the aforementioned dECM hydrogel demonstrates potential for enhancing angiogenesis, the tissue specificity of ECM indicates that ECM derived from highly vascularized tissues may exhibit superior angiogenic activity [26]. We further hypothesize that ECM derived from pathological vascular hyperplastic tissues may retain a unique "angiogenic memory" within its matrix composition and physical architecture, thereby conferring enhanced pro-angiogenic bioactivity. Inspired by this, we propose to prepare a bioactive ECM hydrogel scaffold using human hepatic cavernous hemangioma (HCH) specimens, aiming to construct a microenvironment that recruits endogenous ECs, enhances their function and ultimately stimulates vascular growth. HCH is the most common benign liver tumor. Histologically, it is characterized by cavernous venous malformations resulting from excessive capillary proliferation [27]. The pathogenesis of HCH remains unclear; the prevailing view suggests that abnormal proliferation of vascular ECs during embryonic development, combined with continuous estrogen stimulation, contributes to its onset and progression [28]. As a representative vascular benign tumor, the ECM of HCH is not only enriched with basement membrane proteins (such as collagen IV and laminin) but may also encapsulate specific growth factors and regulatory networks secreted by vascular endothelial cells during their highly active yet organized proliferation [29]. Therefore, we hypothesize that ECM derived from HCH could effectively activate ECs and promote angiogenesis, making it a potential source material for regenerative medicine. The human tissue origin of this material endows it with significant potential for clinical translation. Moreover, the benign nature of HCH implies greater biosafety of HCH dECM hydrogel compared to Matrigel derived from mouse sarcoma.

However, the development and application of ECM hydrogels also face certain challenges; for instance, their mechanical properties can be suboptimal due to unstable non-covalent intermolecular interactions during self-assembly [30,31]. Among various reinforcement strategies, we selected gelatin methacryloyl (GelMA) as a composite material. GelMA is synthesized by functionalizing gelatin with methacryloyl side groups. Upon exposure to ultraviolet light in the presence of a photoinitiator, these groups undergo free-radical polymerization, forming a covalently crosslinked hydrogel network. Compared to synthetic alternatives (e.g., polyethylene glycol diacrylate (PEGDA)), GelMA is collagen-derived, offering superior biocompatibility and intrinsic Arg-Gly-Asp (RGD) motifs [32]. Its photo-crosslinkability also enables precise tuning of mechanical properties and forms a network structure more favorable for cell migration and infiltration than other natural polymers (e.g., alginate) [33]. Thus, GelMA not only provides precisely controllable mechanical support to compensate for the limitations of dECM but also, through its RGD sequence, synergizes with inherent dECM adhesion motifs (e.g., the laminin-derived YIGSR sequence) and bioactive factors to enhance EC adhesion and migration [34]. Therefore, combining HCH dECM with GelMA represents a promising biomaterial strategy for inducing angiogenesis and promoting tissue regeneration.

**Scheme 1:**
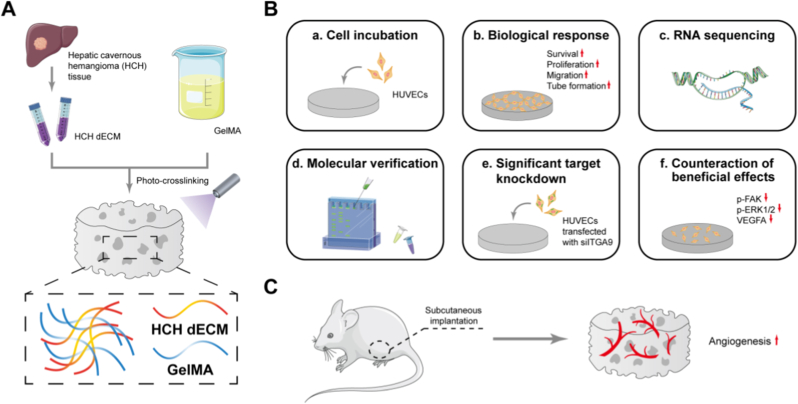
The HCH dECM/GelMA composite hydrogel and its role in promoting angiogenesis. (A) Construction of the HCH dECM/GelMA hydrogel. (B) The hydrogel enhanced the viability, proliferation, migration, and tube formation of endothelial cells via the ITGA9–FAK–ERK1/2 axis. (C) The hydrogel promotes robust angiogenesis in a mouse subcutaneous implantation model.

The aim of this study was to develop a bioactive HCH dECM/GelMA composite hydrogel scaffold to induce rapid and extensive formation of vascular networks. This work also aims to address several common challenges in current dECM research, including improving mechanical stability through compositing with GelMA, as well as exploring a novel, accessible human-derived ECM source for clinical translation. We demonstrated, for the first time worldwide, that the HCH dECM/GelMA composite hydrogel enhances the activity and biological functions of human umbilical vein endothelial cells (HUVECs) and effectively promotes vascularization in vivo. Given the compositional profile of HCH dECM, we postulate that its pro-angiogenic effects may be closely associated with integrin-mediated cell–ECM interactions, potentially involving the activation of downstream signaling pathways such as Focal Adhesion Kinase (FAK)–Extracellular Signal-Regulated Kinase 1/2 (ERK1/2). Further investigations were conducted to explore the underlying molecular mechanisms and assess the biosafety of the scaffold (Scheme 1).

## 2 Materials and methods

### 2.1 Preparation of HCH dECM/GelMA composite hydrogels

Fresh human HCH specimens were obtained from patients diagnosed with HCH who underwent partial hepatectomy at the Department of Hepatobiliary Surgery, The First Hospital of China Medical University. The collection and use of HCH tissues were approved by the Ethics Committee of the First Hospital of China Medical University (approval no. 2024-575-2). All donors were informed of the experimental procedures and provided written informed consent. The decellularization process followed established methodologies [35]. Briefly, healthy liver tissues adjacent to the lesion and fibrotic portions were removed, and the remaining tissue was minced into pieces less than 2 mm in diameter. The tissue fragments were rinsed with 1 M NaCl and PBS for 30 min, homogenized, and centrifuged at 3000 rpm for 5 min. The pellet was treated with 1% Triton X-100 (Solarbio, China) and a mixed solution containing 10^4^ U/mL DNase and 10^4^ U/mL RNase (Solarbio, China) at 37 °C for 6 h each. After treatment, the samples were washed three times with PBS for 10 min each, lyophilized, and stored at −80 °C. The freeze-dried HCH dECM powder was dissolved in a 4 mg/mL pepsin hydrochloride solution (pH 2.0) and stirred at 300 rpm for 24 h. The digested dECM solution was then neutralized and balanced ionically by adding 0.1 M NaOH and 10 × PBS, followed by incubation at 37 °C for 30 min to form the HCH dECM hydrogel. GelMA synthesis was performed as described in previous studies [36]. In brief, 10 g of gelatin (Solarbio, China) was dissolved in 100 mL of PBS under stirring at 50 °C. Then, 8 mL of methacrylate anhydride (MA; Solarbio, China) was added dropwise at 0.2 mL/min and stirred for 2 h. The reaction was terminated by adding 200 mL of preheated PBS. The mixture was dialyzed using 14,000 Da dialysis bags for 5 days and lyophilized to obtain GelMA sponge. A 0.5% (w/v) lithium phenyl-2,4,6-trimethylbenzoylphosphinate (LAP; Orileaf, China) solution was prepared by dissolving 50 mg of LAP in 10 mL of PBS in a 45 °C water bath for 15 min. Next, 1 g of GelMA was added and dissolved by stirring at 65 °C for 1 h. The solution was sterilized through a 0.22 μm filter to yield a 10% (w/v) GelMA solution. HCH dECM solutions at concentrations of 5, 10, 15, and 20 mg/mL were each mixed with an equal volume of 10% (w/v) GelMA solution. The mixtures were exposed to 405 nm ultraviolet light for 20 s to form HCH dECM/GelMA composite hydrogels (5% GelMA with 2.5-10 mg/mL HCH dECM). The detailed composition of the composite hydrogels is summarized in Supplemental Table 1.

### 2.2 Characterization of HCH dECM hydrogels and composite hydrogels

#### 2.2.1 Histological staining

Fresh native HCH and decellularized HCH dECM slices were fixed in 4% (v/v) formaldehyde (Solarbio, China), dehydrated through a graded ethanol and xylene series, and embedded in paraffin. Sections of 5 μm thickness were prepared using a microtome and stained with hematoxylin and eosin (H&E; Solarbio, China) and Masson's trichrome (Solarbio, China). Decellularization efficiency was assessed using an inverted microscope (Nikon TE2000-S, Japan).

#### 2.2.2 Biochemical analysis

DNA content was quantified using the PicoGreen dsDNA Quantification Kit (Solarbio, China). Freeze-dried HCH and HCH dECM powders were treated with lysis buffer (1% SDS, 10 mM EDTA, 100 mM Tris-HCl, 100 mM NaCl, pH 8.0) and digested with Proteinase K (Solarbio, China). Samples and standards were incubated with PicoGreen reagent for 5 min at room temperature in the dark. Fluorescence was measured using a Varioskan LUX multimode microplate reader (Thermo Fisher Scientific, USA) at excitation/emission wavelengths of 488/520 nm.

Total protein content was determined using the BCA Protein Assay Kit (Solarbio, China). Freeze-dried powders were lysed in ice-cold RIPA buffer (Keygen Biotech, China) supplemented with 1% (v/v) Protease Inhibitor Cocktail (GLPBIO, USA) and 1% (v/v) Phosphatase Inhibitor Cocktail (GLPBIO, USA). Homogenization was performed with glass homogenizers, followed by centrifugation at 16,000×*g* for 20 min. Supernatants and standards were incubated with BCA reagent at 37 °C for 30 min, and absorbance was measured at 562 nm using a Multiskan FC microplate reader (Thermo Fisher Scientific, USA).

Collagen content was assessed using the Hydroxyproline (HYP) Content Assay Kit (Solarbio, China). According to the manufacturer's instructions, samples were treated with 6 M hydrochloric acid and assay reagents, and absorbance was measured at 560 nm with an Evolution 60S spectrophotometer (Thermo Fisher Scientific, USA).

#### 2.2.3 Mechanical properties

The mechanical properties of the hydrogels were evaluated using a WDW-100 electronic universal testing machine (Shanghai Precision Instrument Co., Ltd., China). Cylindrical hydrogel samples (d = 15 mm, h = 10 mm) were placed on the testing platform and compressed at a rate of 5 mm/min. Stress data were recorded continuously until 50% deformation was reached. Young's modulus was calculated as the slope of the linear region of the stress-strain curve between 0% and 5% strain. Rheological properties were analyzed using a rotational rheometer (Anton Paar MCR92, Austria). Frequency sweep tests were performed from 1 to 100 Hz at a fixed strain of 1% to measure the storage modulus (G′) and loss modulus (G″).

#### 2.2.4 Scanning electron microscopy (SEM)

The ultrastructure of hydrogels was examined by SEM. Samples were fixed in 2.5% glutaraldehyde for 1 h, dehydrated in a graded ethanol series, and lyophilized. Freeze-dried samples were mounted on specimen stubs and observed under a scanning electron microscope (HITACHI S-4800, Japan) at 20 kV. The diameter and porosity of nanofibrous structures were analyzed using ImageJ software (NIH, USA).

#### 2.2.5 Proteomic analysis

Liquid chromatography-tandem mass spectrometry (LC-MS/MS) was used to identify peptide sequences in HCH dECM. Fresh HCH specimens were obtained from three patients, and dECM was prepared from each. Proteins were extracted using SDT lysis buffer (4% SDS, 100 mM DTT, 100 mM Tris-HCl, pH 8.0) and quantified with the BCA Protein Assay Kit. Protein digestion was performed using the FASP method [37]. Briefly, detergent, DTT, and iodoacetamide in UA buffer were used to block reduced cysteine residues. The protein suspension was digested overnight at 37 °C with trypsin (Promega, USA) at a 50:1 ratio. Peptide mixtures were collected by centrifugation at 16,000×*g* for 15 min and desalted using C18 StageTips. Peptide concentrations were determined by OD280 measurement with a Nanodrop One spectrophotometer (Thermo Fisher Scientific, USA). LC-MS/MS analysis was conducted on an Orbitrap Astral mass spectrometer coupled to a Vanquish Neo UHPLC system (Thermo Fisher Scientific, USA). Data-independent acquisition (DIA) data were processed with DIA-NN 1.8.1 and searched against the UniProtKB database. Results were filtered at a false discovery rate (FDR) < 1% for both peptide-spectrum matches and proteins. Hierarchical clustering and volcano plots were generated using R version 4.3.1. Significantly differentially expressed proteins (DEPs) were identified by Student's t-test (p < 0.05, |log_2_ fold change| ≥ 1). Gene Ontology (GO) and Kyoto Encyclopedia of Genes and Genomes (KEGG) enrichment analyses were performed using Fisher's exact test with FDR correction (p < 0.05).

### 2.3 Cell culture

The human umbilical vein endothelial cell line EA.hy926 was purchased from Pricella Biotechnology Co., Ltd. (Wuhan, China) and cultured in high-glucose DMEM medium (Pricella, China) supplemented with 10% fetal bovine serum (FBS; Pricella, China) and 1% penicillin-streptomycin (Pricella, China) at 37 °C in a 5% CO_2_ incubator (Thermo Fisher Scientific, USA).

### 2.4 Cell viability assay

To assess biocompatibility, HCH dECM/GelMA composite hydrogels with gradient HCH dECM concentrations were prepared in 24-well plates (300 μL/well). HUVECs were seeded onto the hydrogel surfaces or into empty wells (2 × 10^4^ cells/well). After 3 and 7 days of culture, cell viability and morphology were evaluated using a Calcein-AM/PI Live/Dead Cell Double Stain Kit (Solarbio, China). Images were acquired with a fluorescence microscope (KEYENCE BZ-X800, China), and live/dead cells were quantified using ImageJ software.

### 2.5 Cell counting Kit-8 (CCK-8) assay

To evaluate the proliferation-promoting effects, hydrogels were prepared in 96-well plates (50 μL/well). HUVECs were seeded onto the hydrogel surfaces or into empty wells (5 × 10^3^ cells/well). After 1, 2, and 3 days, the culture medium was replaced with 100 μL of fresh medium containing 10% (v/v) CCK-8 reagent (Solarbio, China) and incubated for 2 h at 37 °C. An equal volume of medium from each well was transferred to a new 96-well plate, and absorbance was measured at 450 nm using a microplate reader.

### 2.6 Scratch wound assay

A scratch wound assay was used to assess HUVEC migration. Cells were seeded in 6-well plates at a density of 2 × 10^5^ cells/well and cultured until ∼90% confluent. A linear scratch was created using a 10 μL pipette tip. After washing with PBS, the medium was replaced with serum-free medium containing different concentrations of HCH dECM. Bright-field images were taken at 0, 12, and 24 h, and wound closure was analyzed with ImageJ software.

### 2.7 Transwell migration assay

Cell migration was further evaluated using Transwell chambers (8 μm pore size, Corning, USA). The lower chamber was filled with medium containing different concentrations of HCH dECM. HUVECs resuspended in serum-free medium were seeded into the upper chamber (2 × 10^4^ cells/well). After 24 h, cells that migrated to the lower surface were fixed with 4% paraformaldehyde and stained with 0.1% crystal violet (Solarbio, China). Migrated cells were counted under an inverted microscope using ImageJ.

### 2.8 Tube formation assay

Angiogenesis in vitro was assessed by tube formation assay. Wells of 48-well plates were precoated with 150 μL of GelMA, HCH dECM/GelMA, or Matrigel (Mogengel, China). HUVECs were seeded (2 × 10^4^ cells/well) and incubated for 6, 12, and 24 h. Cells were stained with Calcein-AM, and images were captured with a fluorescence microscope. Total tube length was quantified using ImageJ.

### 2.9 Quantitative real-time PCR (qRT-PCR) analysis

Cells were extracted from hydrogels using GelMA Lysis Buffer (EFL, China) [38]. The lysis buffer was diluted to 0.3 mg/mL with culture medium. Hydrogels loaded with HUVECs were minced and immersed in the lysis buffer at 37 °C for 30 min, followed by centrifugation at 1000 rpm for 5 min to collect cells. Total RNA was extracted with RNAiso (Takara, Japan), reverse-transcribed into cDNA, and amplified with specific primers. qRT-PCR was performed in a 20 μL reaction mixture containing 10 μL TB Green Premix Ex *Taq*II (Takara, Japan), 6 μL nuclease-free H_2_O, 2 μL primer, and 2 μL cDNA. Gene expression levels were normalized to GAPDH. Primer sequences are listed in Supplemental Table 2.

### 2.10 Western blot analysis

Cells were collected, and total protein was extracted and quantified as described. Equal amounts of protein (20 μg) were separated by 10% SDS-PAGE and transferred to PVDF membranes (Keygen Biotech, China). Membranes were blocked with 5% bovine serum albumin (BSA; Keygen Biotech, China) in TBST for 1 h and incubated with primary antibodies overnight at 4 °C. HRP-conjugated secondary antibodies were applied for 1 h at room temperature. Blots were visualized using an enhanced chemiluminescence detection system (Tanon 5200, China). Primary antibodies included: anti-VEGFA (1:10,000, Proteintech, China), anti-PDGF-BB (1:1,000, Proteintech, China), anti-ITGA9 (1:1,000, Proteintech, China), anti-FAK (1:1,000, Proteintech, China), anti-phospho-FAK (1:1,000, Cell Signaling Technology, USA), anti-ERK1/2 (1:10,000, Proteintech, China), anti-phospho-ERK1/2 (1:5,000, Proteintech, China), and anti-β-Tubulin (1:10,000, Proteintech, China).

### 2.11 RNA sequencing and data analysis

HUVECs were cultured on GelMA or HCH dECM/GelMA hydrogels for 48 h. Cells cultured in standard dishes served as controls. Cells were collected and RNA was extracted as described. Libraries were prepared from 1 μg total RNA using the TruSeq Stranded Total RNA Kit with Ribo-Zero Gold (Illumina, USA). Reads were aligned to the human reference genome (GRCh38.p13) using TopHat v1.4.1. Differential gene expression analysis was performed with DESeq2 in R, with adjusted p-values calculated using the Benjamini-Hochberg method. Significantly differentially expressed genes (DEGs) were defined as those with |log_2_ fold change| ≥ 1 and adjusted p-value <0.05. Gene Set Enrichment Analysis (GSEA) was conducted using the clusterProfiler package. Enriched pathways were considered significant at p < 0.05 and normalized enrichment score (NES) > 1. GO and KEGG enrichment analyses were performed as described above.

### 2.12 Small interfering RNA (siRNA) transfection

Integrin alpha 9 (ITGA9) knockdown was achieved using siRNAs designed and synthesized by Tsingke Biotechnology Co., Ltd. (Beijing, China). Sequences are provided in Supplemental Table 3. EA.hy926 cells were transfected with 40 nmol/L siRNA using Lipofectamine 3000 (Thermo Fisher Scientific, USA) according to the manufacturer's protocol. Cells were harvested 72 h post-transfection for subsequent experiments.

### 2.13 In vivo angiogenesis assessment

Male C57BL/6J mice (20–25 g, specific pathogen-free) were obtained from the Laboratory Animal Resource Center of Liaoning Province. All animal procedures were approved by the Ethics Committee of the First Hospital of China Medical University (approval no. 2024-575-2). Forty-eight mice were randomly divided into 4 groups: GelMA, HCH gel, Liver gel, and PL gel. Liver gel (liver dECM/GelMA composite hydrogel) and PL gel (placental dECM/GelMA composite hydrogel) were fabricated from healthy liver tissue adjacent to HCH lesions and human placental tissues, respectively, following the same protocol used for the HCH dECM/GelMA. [30,39]. The use of human liver and placental specimens was also ethically approved. Under isoflurane anesthesia, a 2 cm midline abdominal incision was made, and subcutaneous connective tissue on the left side was bluntly dissected. A cylindrical hydrogel (d = 8 mm, h = 2 mm) was implanted into the subcutaneous space, and the skin was sutured. Half of the mice in each group (n = 6) were euthanized at 2 weeks post-implantation, and hydrogels with surrounding tissues were harvested for histological analysis. The remaining mice (n = 6) were euthanized at 4 weeks post-implantation. Similarly, the hydrogels with surrounding tissues were harvested for histological analysis, and tissues from major organs were also collected for biosafety assessment.

### 2.14 Histological analysis and immunostaining

Tissue sections were prepared and stained with H&E as described. For immunofluorescence, slides were deparaffinized, rehydrated, subjected to antigen retrieval and blocked with 3% BSA in PBS for 30 min. Sections were then incubated overnight at 4 °C with primary antibodies against CD31 (1:100, Abcam, UK) and α-smooth muscle actin (α-SMA; 1:500, Servicebio, China). After washing, slides were incubated with species-specific secondary antibodies conjugated to Cy3 or FITC (Servicebio, China) for 1 h at 37 °C. Nuclei were counterstained with DAPI (Servicebio, China). For immunohistochemistry, after deparaffinization and rehydration, antigen retrieval was performed followed by blocking of endogenous peroxidase activity. Sections were blocked with 3% BSA for 30 min, then incubated overnight at 4 °C with anti-ITGA9 (1:200, Proteintech, China), followed by incubation with an HRP-conjugated goat anti-rabbit secondary antibody (Servicebio, China) for 1 h at room temperature. Signal was developed using a diaminobenzidine chromogen substrate (MXB, China), and nuclei were counterstained with hematoxylin. All images were acquired using a fluorescence/bright-field microscope and analyzed semi-quantitatively with ImageJ software.

### 2.15 Statistical analysis

Data are presented as mean ± standard deviation. All experiments were performed independently with at least three technical replicates per group. Differences between groups were analyzed using a two-tailed Student's t-test or one-way ANOVA. A p-value <0.05 was considered statistically significant.

## 3 Results

### 3.1 Construction and characterization of HCH dECM/GelMA composite hydrogels

**Fig. 1:**
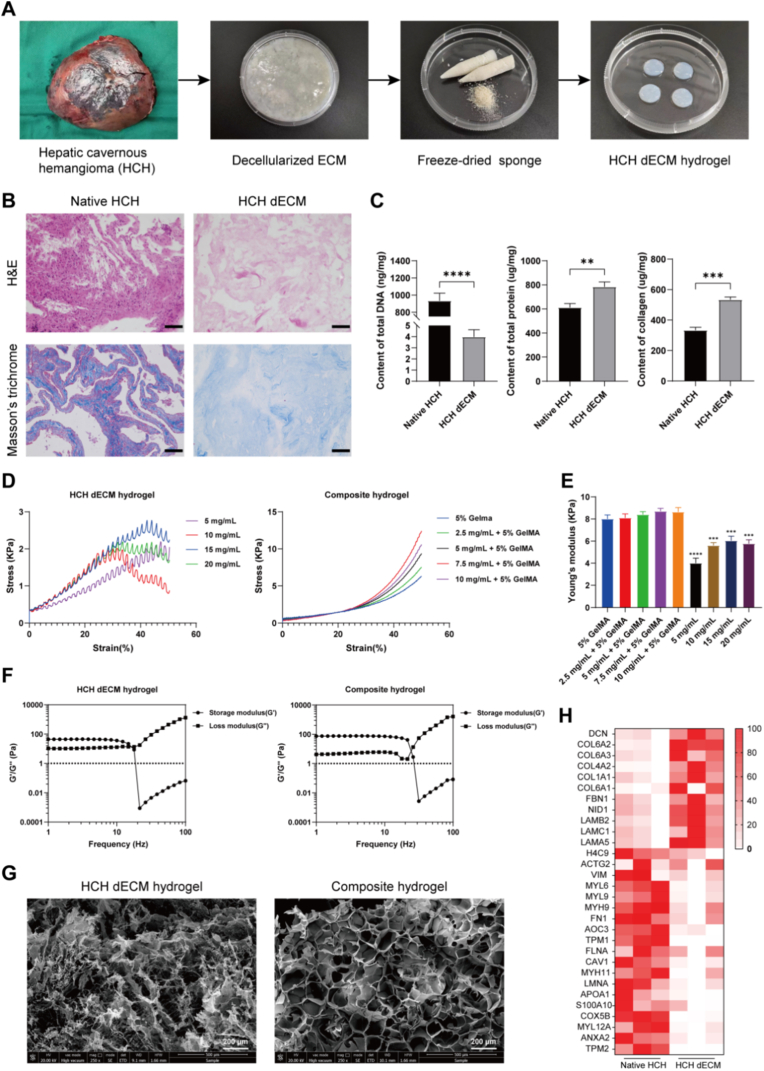
Construction and characterization of HCH dECM and HCH dECM/GelMA composite hydrogels. (A) The preparation process of HCH dECM hydrogel, including decellularization of HCH tissues, freeze-drying, milling, pepsin digestion, and sol-gel transition. (B) H&E and Masson's Trichrome staining of native HCH tissue and HCH dECM (scale bars = 100 μm). (C) Quantitative analysis of residual DNA, total protein, and collagen content in native HCH and HCH dECM. (D) Compressive stress-strain curves. (E) Young's modulus derived from graph D. (F) Frequency-dependent modulus scans of HCH dECM hydrogel (10 mg/mL) and the composite hydrogel (5 mg/mL + 5% GelMA). (G) SEM micrographs showing the ultrastructure of HCH dECM hydrogel (10 mg/mL) and the composite hydrogel (5 mg/mL + 5% GelMA) (scale bars = 200 μm). (H) Clustered heatmap of the top 30 significantly differentially expressed proteins between native HCH and HCH dECM. (n = 3, ∗∗p < 0.01, ∗∗∗p < 0.001, ∗∗∗∗p < 0.0001).

The preparation process of the HCH dECM hydrogel, involving decellularization of HCH tissues, freeze-drying, milling, pepsin digestion, and sol-gel transition, is depicted in Fig. 1A. H&E and Masson's Trichrome staining confirmed the complete removal of cellular components post-decellularization, while extracellular matrix components were preserved (Fig. 1B). Quantitative assessment of residual DNA content further verified effective decellularization, with HCH dECM containing 4.0 ± 0.6 ng/mg, significantly lower than the 933.3 ± 89.0 ng/mg in native HCH tissue. Biochemical quantification revealed that, compared to an equivalent mass of native HCH tissue, HCH dECM contained significantly higher total protein and collagen contents (146.8% and 183.4%, respectively) (Fig. 1C).

Mechanical property assessment via stress-strain curves showed that all HCH dECM hydrogels fractured at approximately 40% deformation, with maximum stress below 3 KPa. In contrast, 5% (w/v) GelMA hydrogel and HCH dECM/GelMA composite hydrogels maintained integrity and elasticity up to 50% deformation, withstanding stresses around 10 KPa (Fig. 1D). Young's modulus calculations indicated a gradual increase with rising HCH dECM concentration, peaking at 6.0 ± 0.4 KPa at 15 mg/mL before declining. All values remained significantly lower than those of GelMA and composite hydrogels. The composite hydrogels exhibited a concentration-dependent trend in Young's modulus similar to HCH dECM hydrogels; however, the difference was minimal and not statistically significant compared to GelMA (8.0 ± 0.4 KPa), suggesting that GelMA is the primary contributor to the mechanical strength of the composite hydrogel (Fig. 1E). Rheological analysis revealed that composite hydrogels, compared to pure HCH dECM hydrogels, displayed a significantly higher storage modulus (G′) and lower loss modulus (G″) in the gel state, along with a higher frequency threshold for transition to a fluid state (Fig. 1F). This indicates superior stability of the composite hydrogel, whereas the gel state of HCH dECM hydrogel is less stable, with intermolecular forces easily disrupted by external influences. SEM imaging showed that composite hydrogels possessed more uniform micropore size and distribution compared to HCH dECM hydrogels (Fig. 1G). For instance, the pore size and porosity of 10 mg/mL HCH dECM hydrogel were 97.1 ± 44.0 μm and 58.0% ± 5.0%, respectively, significantly lower than the 170.2 ± 18.8 μm pore size and 76.4% ± 4.6% porosity of the corresponding composite hydrogel (Supplemental Fig. 1A). These findings demonstrate that incorporating GelMA improves the suboptimal mechanical properties of HCH dECM hydrogels.

Proteomic analysis via volcano plot identified 197 upregulated and 1543 down-regulated proteins in HCH dECM compared to native HCH (Supplemental Fig. 1B). A clustered heatmap displayed the top 30 DEPs. Upregulated proteins in HCH dECM included multiple basement membrane components, such as type IV collagen, Nidogen-1, and Laminin-521 (Fig. 1H). GO enrichment analysis indicated that DEPs were significantly enriched in biological processes including cell adhesion and protein folding, as well as in cellular components interacting with ECM, and molecular functions such as ECM binding, integrin binding, and cell adhesion molecule binding (Supplemental Fig. 1C). KEGG enrichment analysis revealed DEP enrichment in ECM-related pathways, including focal adhesion, ECM-receptor interaction, and cell adhesion molecules (Supplemental Fig. 1D). These results elucidate the fundamental material basis underlying the angiogenic activity of HCH dECM. For instance, Nidogen-1 directly binds growth factors, protecting them from degradation and facilitating their presentation to EC receptors, thereby precisely regulating angiogenic signaling [40]. Laminin critically directs ECs to establish apicobasal polarity and assemble into mature, lumenized blood vessels during vascular maturation, modulating cell polarity and apoptosis via interactions with integrins and dystroglycan [41].

### 3.2 Biological response of HUVECs to HCH dECM

**Fig. 2:**
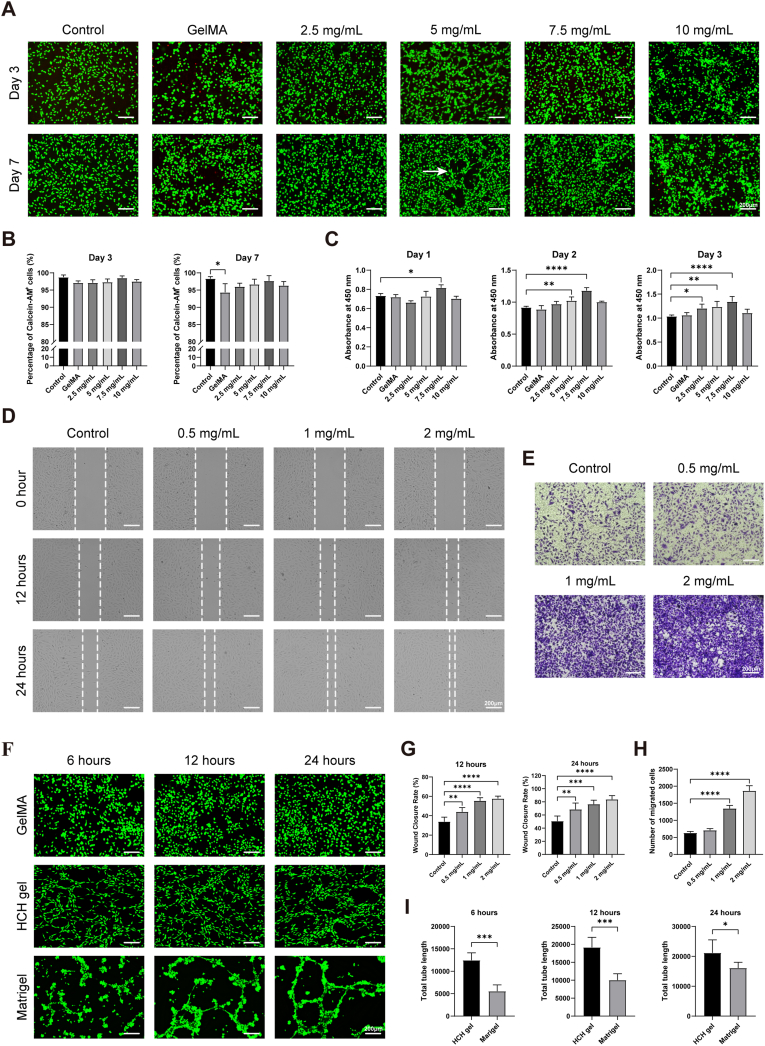
Biological responses of HUVECs to HCH dECM. (A) Live/dead staining of HUVECs cultured on HCH dECM/GelMA composite hydrogels (HCH gel) with varying dECM concentrations (scale bars = 200 μm). (B) Quantification of cell viability from graph A. (C) CCK-8 assay assessing HUVEC proliferation in response to HCH gel. (D) Scratch wound assay demonstrating HUVEC migration (scale bars = 200 μm). (E) Transwell migration assay further evaluating HUVEC migration (scale bars = 200 μm). (F) Tube formation assay assessing the angiogenic potential of HUVECs (scale bars = 200 μm). (G) Wound closure rate quantified from graph D. (H) Number of migrating cells quantified from graph E. (I) Total tube length of vessel-like structures quantified from graph F. (n = 5, ∗p < 0.05, ∗∗p < 0.01, ∗∗∗p < 0.001, ∗∗∗∗p < 0.0001).

We first evaluated the in vitro biocompatibility of the HCH dECM/GelMA composite hydrogel (hereafter referred to as HCH gel) using live/dead cell double staining (Fig. 2A). Results showed a gradual increase in live cell proportion with rising HCH dECM concentration, reaching a maximum of 97.7% ± 1.5% at 7.5 mg/mL, though intergroup differences were not statistically significant. After 7 days of incubation, this trend became more pronounced. The live cell proportion in the GelMA group (94.3% ± 2.5%) was significantly lower than in the control group (98.3% ± 0.6%), while no significant difference was observed between HCH gel groups and the control (Fig. 2B). Additionally, HUVECs cultured on 5 mg/mL HCH gel formed vessel-like connections similar to those on Matrigel, indicating that HCH gel not only supports HUVEC viability but also promotes their polarization and tube formation.

CCK-8 assay assessed the effect of HCH gel on HUVEC proliferation. HCH gel significantly enhanced HUVEC proliferation on days 1, 2, and 3 of incubation (Fig. 2C). The most pronounced effect was observed with 7.5 mg/mL HCH gel, consistent with the concentration-dependent trend in biocompatibility. On day 3, this concentration yielded the highest optical density (OD) value (1.35 ± 0.11 vs. 1.04 ± 0.03 in the control group).

Scratch wound assay revealed that HUVECs exhibited significantly enhanced migration in culture medium supplemented with gradient concentrations of HCH dECM solution (Fig. 2D and G). Transwell migration assay showed substantially more cells migrated to the lower chamber in the 1 mg/mL and 2 mg/mL HCH dECM groups (1346.0 ± 89.5 and 1866.0 ± 150.6 cells/field, respectively) compared to the control group (632.0 ± 41.1 cells/field) (Fig. 2E and H).

Given the observation of vessel-like structures when HUVECs were cultured on 5 mg/mL HCH gel, a tube formation assay was performed. Reticular connections formed 6 h post-seeding, with tube-like structures evident after 24 h (Fig. 2F). Total tube lengths in the HCH gel group at 6, 12, and 24 h (12.5 ± 1.7 μm, 19.2 ± 2.8 μm, and 21.2 ± 4.3 μm, respectively) were significantly greater than those in the Matrigel group (5.5 ± 1.2 μm, 10.0 ± 1.8 μm, and 16.1 ± 1.9 μm, respectively) (Fig. 2I). HUVECs on Matrigel tended to aggregate into clusters, forming thick-walled tubular structures with distinct boundaries but fewer branches. In contrast, HUVECs on HCH gel were more evenly distributed, developing vascular structures with more branches and intricate interconnections, representing a substantial advantage. Although the 7.5 mg/mL HCH gel exhibited marginally higher biocompatibility and supported cell proliferation, the 5 mg/mL gel consistently and markedly enhanced tube formation—a hallmark of EC activation and functional assembly. This functional advantage was considered biologically much more significant. Therefore, the 5 mg/mL HCH gel was selected as the optimal concentration for subsequent studies.

### 3.3 In vivo angiogenic effects, degeneration and biocompatibility of HCH gel

**Fig. 3:**
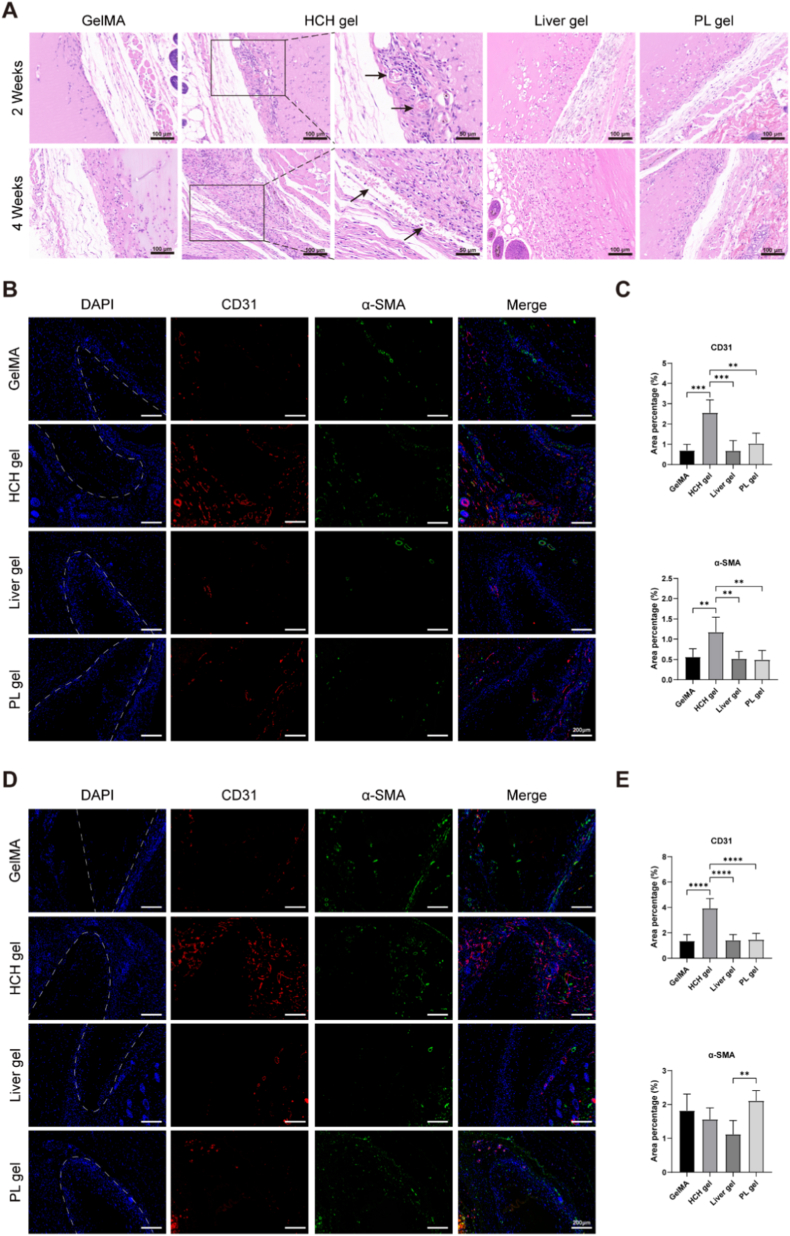
In vivo angiogenic effects of HCH gel. (A) H&E staining of tissue sections showing the angiogenic effect after 4 weeks of hydrogel implantation. (B) Immunofluorescence co-staining for CD31 and α-SMA assessing vascular proliferation at 2 weeks post-implantation (scale bars = 200 μm). White dotted lines indicate the hydrogel–tissue interface. (C) Quantification of the CD31^+^ and α-SMA^+^ area from graph B. (D) Immunofluorescence co-staining for CD31 and α-SMA at 4 weeks post-implantation (scale bars = 200 μm). (E) Quantification of the CD31^+^ and α-SMA^+^ area from graph D. (n = 6, ∗p < 0.05, ∗∗p < 0.01, ∗∗∗p < 0.001, ∗∗∗∗p < 0.0001).

Based on the comprehensive in vitro effects of different HCH gel concentrations on HUVEC behavior, 5 mg/mL HCH gel was implanted into the mouse abdominal subcutaneous space to further assess its pro-angiogenic role in vivo. H&E staining indicated capillary induction and migration onto HCH gel surfaces post-implantation, whereas no obvious vascular formation was observed in the GelMA, Liver gel or PL gel groups (Fig. 3A). 2 weeks post-implantation, circular vascular cross-sections with disc-shaped red blood cells was observed superficially within the HCH gel. After 4 weeks, elongated blood vessels extended across the HCH gel surface. Additionally, cell infiltration into the HCH gel was significantly higher compared to GelMA, Liver gel and PL gel, further suggesting HCH gel promotes cell migration in vivo.

Immunofluorescence staining for CD31 and α-SMA further evaluated in vivo angiogenesis. Capillaries extensively proliferated at the HCH gel–tissue interface (Fig. 3B and D). The CD31-positive area percentages in the HCH gel group at 2 and 4 weeks post-implantation (2.6% ± 0.6% and 3.9% ± 0.8%, respectively) were significantly higher than in the GelMA (0.7% ± 0.3% and 1.4% ± 0.5%), Liver gel (0.7% ± 0.5% and 1.4% ± 0.4%) and PL gel groups (1.0% ± 0.5% and 1.5% ± 0.5%). The α-SMA-positive area in the HCH gel group (1.2% ± 0.4%) was significantly higher than in the GelMA (0.6% ± 0.2%), Liver gel (0.5% ± 0.2%) and PL gel groups (0.5% ± 0.2%) at 2 weeks, though no significant differences were detected at 4 weeks (Fig. 3C and E). These findings suggest that HCH gel significantly promotes angiogenesis and facilitates early blood vessel maturation, even in poorly vascularized tissues like subcutaneous tissue.

To evaluate in vivo degradation, the maximum diameter of the hydrogels was measured (Supplemental Fig. 2A). In the HCH gel group, the maximum diameters were 7.1 ± 0.2 mm at 2 weeks and 6.6 ± 0.2 mm at 4 weeks post-implantation (Supplemental Fig. 2B). The degradation profiles indicated that the HCH gel degraded slightly slower than GelMA, while area-under-the-curve analysis revealed no significant difference among the groups (Supplemental Fig. 2C). Additionally, H&E staining of heart, liver, spleen, lung, and kidney showed that HCH gel exhibited good biocompatibility, with staining results largely comparable to those of the control mice, indicating no significant damage to vital organs during at least 4 weeks of implantation (Supplemental Fig. 3).

### 3.4 HCH gel enhances expression of angiogenic cytokines and pathways in HUVECs

**Fig. 4:**
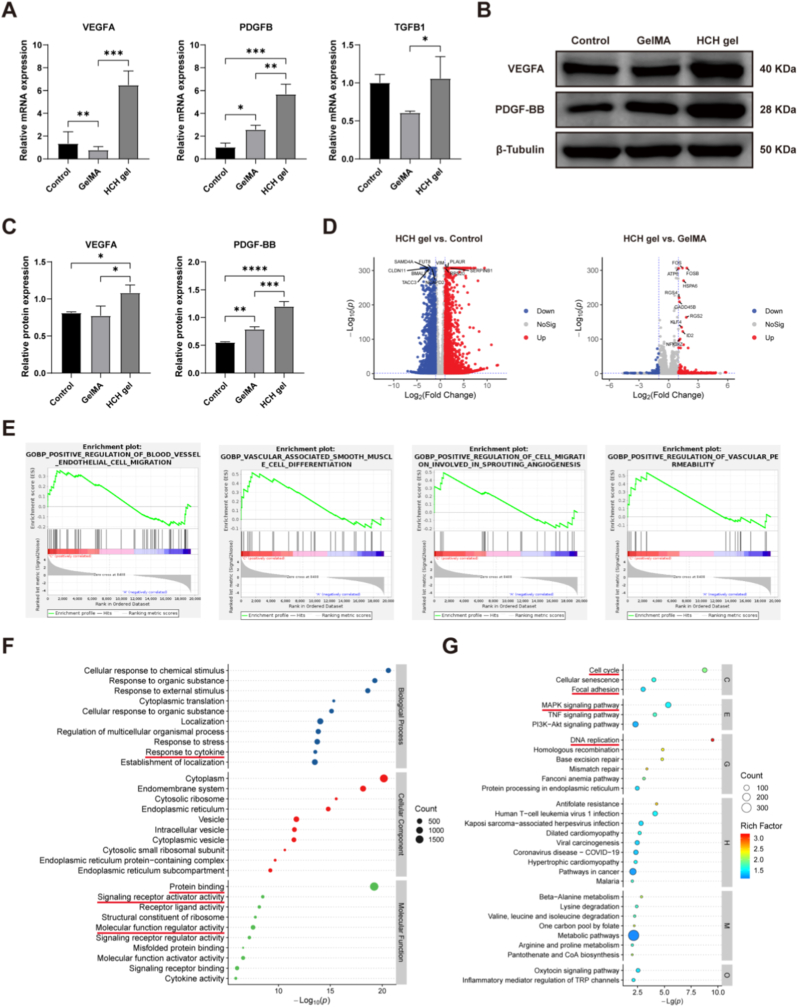
Enhancement of angiogenic cytokine expression and signaling pathways in HUVECs by the HCH gel. (A) mRNA expression levels of VEGFA, PDGFB, and TGFB1 in HUVECs measured by qRT-PCR. (B) Protein expression of VEGFA and PDGF-BB assessed by Western blot. (C) Quantification of Western blot results. (D) Volcano plots showing significantly differentially expressed genes in the HCH gel group compared to the control and GelMA groups. (E) GSEA of angiogenic signaling pathways regulated by the HCH gel. (F) GO enrichment analysis. (G) KEGG pathway enrichment analysis. (n = 3, ∗p < 0.05, ∗∗p < 0.01, ∗∗∗p < 0.001, ∗∗∗∗p < 0.0001).

To investigate the mechanisms underlying HCH gel's pro-angiogenic effects, qRT-PCR analysis assessed variations in angiogenic cytokine expression. VEGFA and platelet-derived growth factor B (PDGFB) expression levels were significantly elevated in the HCH gel group compared to the control and GelMA groups. HCH gel also enhanced transforming growth factor beta 1 (TGFB1) expression relative to GelMA, though no significant difference was observed compared to the control (Fig. 4A). Western blot analysis confirmed enhanced VEGFA and platelet-derived growth factor-BB (PDGF-BB) protein expression in the HCH gel group (Fig. 4B and C). VEGFA is a critical driver of angiogenesis initiation and elongation, while PDGF-BB is essential for vessel maturation, stability, and functionalization [42]. The synergistic action of these factors facilitates EC connection into a complete vascular network.

RNA sequencing was performed to identify pathways activated in HUVECs cultured on HCH gel. Volcano plots identified 4262 DEGs between the HCH gel and control groups, and 314 DEGs between the HCH gel and GelMA groups (Fig. 4D). GSEA revealed significant enrichment of angiogenesis-related pathways in the HCH gel group versus control, including positive regulation of blood vessel endothelial cell migration, vascular smooth muscle cell differentiation, positive regulation of cell migration involved in sprouting angiogenesis, and positive regulation of vascular permeability (Fig. 4E). GO enrichment analysis showed significant upregulation of biological processes related to cytokine response, and molecular functions including protein binding, signaling receptor activator activity, and molecular function regulator activity (Fig. 4F). KEGG enrichment analysis identified pathways such as cell cycle, DNA replication, focal adhesion, and mitogen-activated protein kinase (MAPK) signaling (Fig. 4G). Similarly, GO and KEGG analyses of DEGs between HCH gel and GelMA groups revealed enrichment in pathways including cell communication, signal receptor binding, cell cycle, cell adhesion molecules, and MAPK signaling (Supplemental Fig. 4A and B). The MAPK signaling pathway crucially regulates EC vitality and behavior. Furthermore, integrin binding and FAK pathways, identified in proteomic analysis, are significant upstream molecular interactions activating the MAPK cascade [43]. Thus, we propose that HCH gel promotes HUVEC proliferation, migration, and tube formation via the integrin-FAK-MAPK signaling axis.

### 3.5 HCH gel promotes angiogenesis via the ITGA9-FAK-ERK1/2 axis

**Fig. 5:**
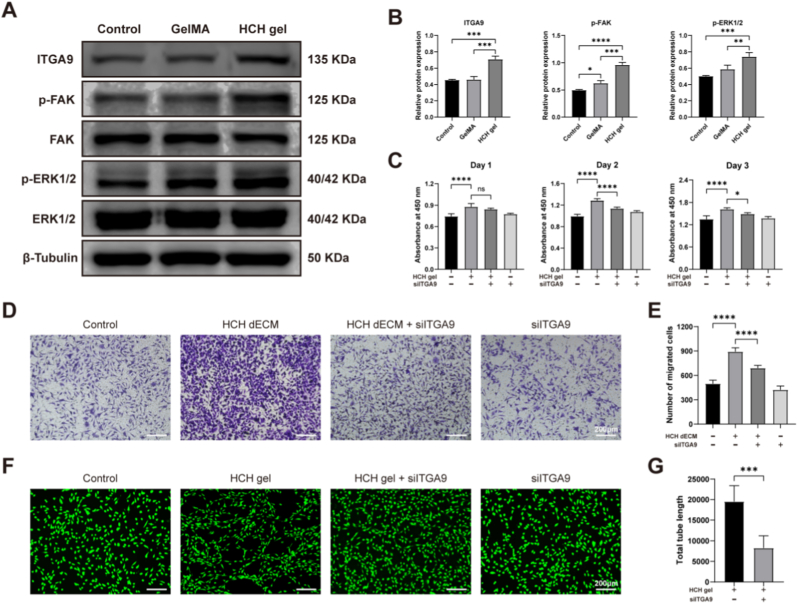
Mechanism underlying the regulation of HUVEC behavior by the HCH gel. (A) Western blot analysis of ITGA9 expression and the phosphorylation status of FAK and ERK1/2. (B) Quantification of Western blot results (n = 3). (C) CCK-8 assay detecting the effect of ITGA9 knockdown (KD) on the proliferation of HUVECs cultured on the HCH gel (n = 5). (D) Transwell migration assay evaluating the effect of ITGA9 KD on HUVEC migration (scale bars = 200 μm). (E) Quantification of the number of migrating cells from graph D (n = 5). (F) Tube formation assay assessing the effect of ITGA9 KD on the angiogenic capacity of HUVECs (incubation for 12 h, scale bars = 200 μm). (G) Quantification of the total tube length from graph F (n = 5). (∗p < 0.05, ∗∗p < 0.01, ∗∗∗p < 0.001, ∗∗∗∗p < 0.0001).

To clarify the specific molecular mechanism, DEGs were filtered with |log_2_ Fold Change| ≥ 2. Intersection analysis of DEGs from HCH gel vs. control and HCH gel vs. GelMA comparisons identified ITGA9 as the sole gene encoding a protein belonging to the integrin family (Supplemental Fig. 4C). Western blot analysis confirmed significantly higher ITGA9 expression in HUVECs cultured on HCH gel compared to those on plates or GelMA. Phosphorylation levels of FAK and ERK1/2 were also significantly elevated in the HCH gel group (Fig. 5A and B). The ERK1/2 pathway, a primary MAPK signaling cascade component, promotes EC proliferation and migration. ERK1/2 upregulation enhances synthesis of transcription factor complexes like AP-1, which directly influence cellular behavior by regulating cell cycle and cytoskeletal proteins, or via upregulation of pro-angiogenic growth factors. Conversely, VEGFA-receptor binding can also activate ERK1/2. This positive feedback loop creates a regulatory network for rapid neovascularization [44].

To determine whether ITGA9 knockdown (KD) counteracts the beneficial effects of HCH gel, HUVECs were transfected with ITGA9-specific siRNA (siITGA9) or a non-targeting control siRNA (siNC) prior to seeding on the gel. Knockdown efficiency was confirmed by qRT-PCR and Western blot, with one siRNA (si-3) exhibiting the highest efficiency at both the RNA and protein levels (Supplemental Fig. 5). CCK-8 assay indicated that the OD value in the HCH gel/siITGA9 group was significantly lower than in the HCH gel/siNC group on days 2 and 3 (Fig. 5C). Transwell migration assay showed significantly fewer migrated cells in the HCH dECM/siITGA9 group (690.2 ± 33.9 cells/field) compared to the HCH dECM/siNC group (891.0 ± 48.1 cells/field) (Fig. 5D and E). Total tube length formed by ITGA9 KD HUVECs (8.3 ± 3.0 μm) was significantly shorter than that of HUVECs cultured directly on HCH gel (19.5 ± 3.9 μm) (Fig. 5F and G). Notably, ITGA9 KD alone did not significantly alter HUVEC biological behaviors compared to the control, further substantiating that ITGA9 exerts substantial effects primarily in the presence of HCH dECM. These results underscore ITGA9 as a critical mediator of HCH dECM's pro-angiogenic effects.

**Fig. 6:**
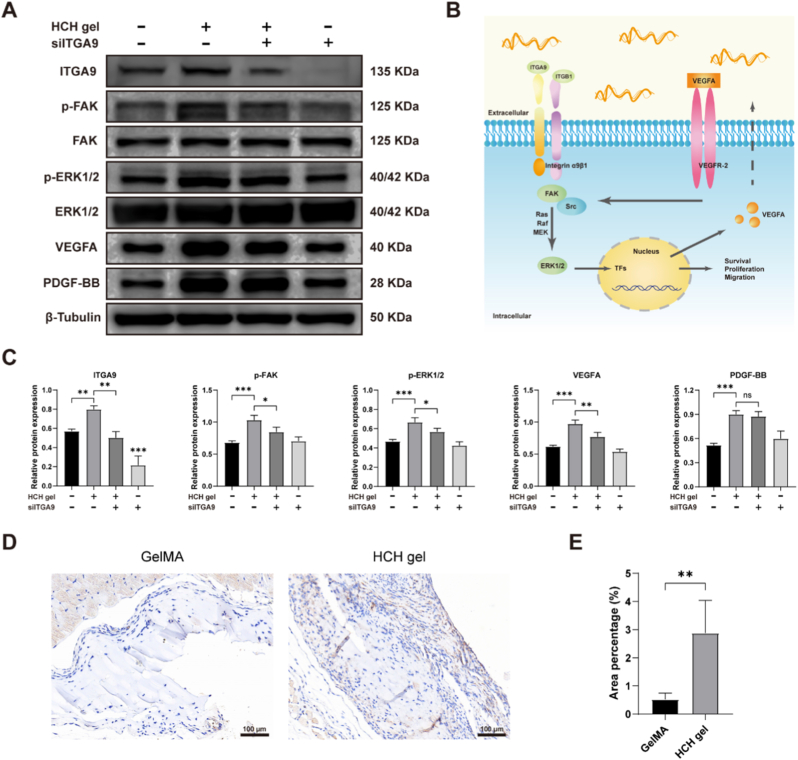
Mechanism of angiogenesis promotion by the HCH gel. (A) Western blot analysis of the effect of ITGA9 KD on the phosphorylation of FAK and ERK1/2, and the expression of VEGFA and PDGF-BB in HUVECs cultured on the HCH gel. (B) Proposed signaling pathway involving ITGA9, FAK, ERK1/2, and VEGFA. (C) Quantification of Western blot results from graph A (n = 3). (D) Immunohistochemical staining of ITGA9 expression at the interface between the HCH gel and surrounding tissue (scale bars = 100 μm). (E) Quantification of the ITGA9-positive area from graph D (n = 6). (∗p < 0.05, ∗∗p < 0.01, ∗∗∗p < 0.001).

We further investigated expression levels of downstream pathways and angiogenic factors following ITGA9 KD. Western blot analysis showed that phosphorylation levels of FAK and ERK1/2 in HUVECs cultured on HCH gel were significantly inhibited by ITGA9 KD. VEGFA expression was also significantly down-regulated, whereas PDGF-BB showed no obvious variation (Fig. 6A and C). ITGA9 KD alone had no significant effect on downstream pathways or angiogenic cytokines, consistent with its minimal impact on HUVEC behavior. Immunohistochemical staining showed ITGA9-positive areas predominantly at the HCH gel–tissue interface, colocalizing with regions of new capillary proliferation (Fig. 6D). The ITGA9-positive area percentage in the HCH gel group (2.9% ± 1.2%) was significantly higher than in the GelMA group (0.5% ± 0.2%) (Fig. 6E). These results indicate that HCH gel promotes angiogenesis via the ITGA9-FAK-ERK1/2 signaling axis (Fig. 6B).

## 4 Discussion

Vascularization plays a crucial role in tissue engineering and regenerative medicine by facilitating the diffusion of oxygen and nutrients [45]. In any living tissue, cells must reside within 100–200 μm of a blood vessel to survive [46]. Without an integrated vascular network, engineered tissues exceeding this diffusion limit develop a necrotic core, leading to cell death and eventual implant failure [47]. However, inducing the formation of functional vascular networks around target tissues remains a major challenge. Although some progress has been made through the delivery of angiogenic cytokines or ECs, these agents often fail to persist stably and function effectively in vivo, resulting in inefficient angiogenesis [48]. These findings suggest that establishing a supportive microenvironment for vascular proliferation may represent a fundamental strategy for promoting angiogenesis.

The ECM provides the structural and biochemical foundation for cell survival and activity, consisting of a complex three-dimensional network of various functional biological macromolecules. These macromolecules interact with cell surface receptors to transmit intricate biological signals and support diverse cellular processes [49]. Moreover, the ECM of different tissues exhibits unique biochemical compositions, physical architectures, and mechanical properties, which provide precise cues to guide cell behavior and ensure tissue-specific functions [50]. Therefore, replicating the native ECM structure of a specific tissue remains the gold standard for designing biomimetic materials. ECM hydrogels, derived from decellularized tissues, retain tissue-specific ECM components and can reassemble into a native-like structure through self-assembly, making them an excellent choice for simulating the natural cellular microenvironment [51]. Inspired by this, we used HCH specimens to prepare a bioactive dECM hydrogel scaffold, aiming to enhance capillary proliferation and migration toward the scaffold, thereby promoting tissue regeneration and supporting the survival and function of delivered cells.

HCH is a vascular mass characterized by excessive capillary proliferation, suggesting it is rich in growth factors and structural macromolecules associated with angiogenesis. As a benign tumor, HCH maintains active yet organized vascular proliferation, unlike the disorganized and uncontrolled expansion seen in malignant tumors, making it a promising biomedical resource [52]. We extracted the ECM from HCH via decellularization and digested it to form an HCH dECM hydrogel. As expected, proteomic analysis confirmed that HCH dECM is highly enriched in various basement membrane proteins and signaling molecules involved in angiogenesis regulation, such as collagen VI, fibrillin, and heparan sulfate proteoglycans. These proteins not only interact with vascular cells but also serve as reservoirs for cytokines. They bind pro-angiogenic cytokines via specific motifs such as the RGD sequence, protecting them from degradation, aiding in spatial organization and concentration gradient formation, and finely regulating their release and activity [53]. These properties enable HCH dECM hydrogels to overcome limitations of single-factor delivery strategies—such as simplistic signaling, poor spatiotemporal control, and immature vascular function.

However, like most native tissue-derived ECM hydrogels, HCH dECM hydrogels exhibit suboptimal mechanical properties. The mechanical integrity of ECM hydrogels primarily depends on type I collagen. Although individual collagen fibers possess high strength, in hydrogels they are thinner, randomly oriented, and connected mainly through weak physical interactions (e.g., hydrogen bonds and hydrophobic interactions) and few covalent bonds [54]. Nevertheless, appropriate scaffold stiffness is critical for supporting cellular behavior, especially in ECs. Zhao et al. demonstrated that hydrogels with intermediate plasticity maximize EC invasion distance and branching sprouting in spheroid models [55]. Therefore, we blended GelMA with HCH dECM to form a composite hydrogel. During photocrosslinking, GelMA forms stable covalent networks, compensating for the mechanical weakness of HCH dECM. Moreover, the uniform and loose microporous structure of GelMA replaces the disordered collagen network in pure HCH dECM hydrogels, offering improved structural support for EC growth and infiltration. Mechanical testing confirmed that the composite hydrogel exhibits a significantly higher Young's modulus, more stable gelation, and more uniform porosity. We opted not to use other stiffening strategies, such as direct methacrylation, due to the compositional complexity of ECM hydrogels, which can lead to inconsistent modification efficiency and potential damage to functional proteins [30]. In summary, the combination of HCH dECM and GelMA provides a synergistic platform, respectively contributing biological activity and structural support for angiogenesis.

We next evaluated the biological response of HUVECs to the HCH gel. As anticipated, the HCH gel significantly enhanced HUVEC viability, proliferation, and migration. Notably, HUVECs cultured on the HCH gel formed vessel-like structures—a hallmark of endothelial activation. Although many substances have been reported to promote angiogenesis, very few materials can induce HUVECs to form such connections under Matrigel-free conditions [56]. Currently, Matrigel, a basement membrane extract from Engelbreth-Holm-Swarm mouse sarcoma, is considered the gold standard for in vitro angiogenesis studies due to its rich content of ECM components and growth factors. However, its batch-to-batch variability, animal-derived nature, and fixed mechanical properties limit its utility in controlled regenerative medicine. Encouragingly, the HCH gel not only supported the formation of complex vascular networks comparable to those on Matrigel, but also showed statistically superior performance in key morphological parameters such as tubule length. This strongly suggests that the HCH gel may serve as a high-performance, human-derived alternative with lower immunogenicity, greater biosafety, and higher clinical translation potential.

Consistent with in vitro results, HCH gel implants in the mouse subcutaneous space robustly promoted angiogenesis. It is important to note that the HCH gel did not carry any exogenous vascular cells or growth factors; its pro-angiogenic effect relied entirely on recruiting host-derived endogenous ECs and modulating their behavior. In vivo angiogenesis is a multi-step process orchestrated by endogenous cells [57]. Our scaffold mimics this natural sequence: it first recruits circulating endothelial progenitor cells or local ECs via inflammatory cues, then guides their behavior through its physicochemical properties and built-in biochemical signals, promoting proliferation, migration, and ultimately lumen formation. Unlike top-down delivery of high-dose growth factors, our strategy empowers host cells from the bottom up, allowing them to secrete necessary factors in a context-dependent manner under local microenvironment guidance. This endogenous signaling network is more refined, coordinated, and efficient than single-factor supplementation, potentially leading to more mature and stable functional vessels. Notably, Matrigel was not employed as a positive control in vivo. This choice was based on the following considerations: Matrigel is derived from malignant mouse sarcoma tissue, and its long-term implantation may pose tumorigenic risks, raising ethical and safety concerns. Additionally, Matrigel degrades rapidly in vivo and lacks sufficient mechanical stability, making it unsuitable for long-term studies of angiogenesis. Therefore, to better meet clinical application needs, we selected PL gel as the control.

To explore the molecular mechanism underlying the pro-angiogenic effect of the HCH gel, we used proteomic and transcriptomic analyses and identified involvement of integrin-related signaling pathways. Integrins are a large family of heterodimeric transmembrane receptors, each composed of an α and a β subunit [58]. They interact with ECM components such as collagen, laminin, and fibronectin to mediate cell–cell and cell–matrix adhesion. Upon activation, integrins cluster into focal adhesions, enhancing FAK phosphorylation and triggering downstream cascades such as MAPK and PI3K–AKT. These pathways activate transcription factors, regulate the cell cycle and cytoskeletal reorganization, and thereby promote EC proliferation and migration [59]. In KEGG enrichment analysis, the MAPK pathway ranked highest in Environmental Information Processing, and the ERK1/2 branch is the most relevant to EC behavior. Thus, we propose that the HCH gel promotes angiogenesis via the ITGA9–FAK–ERK1/2 axis. This was supported by western blot analysis and by altered HUVEC behavior upon ITGA9 KD when cultured on the HCH gel.

ITGA9 encodes the α9 subunit of integrin α9β1, one of the less-studied integrins, though its relevance is increasingly recognized. Existing studies indicate that ITGA9 is important in embryonic vascular development and pathological angiogenesis [60]. Wang et al. showed that nanoparticle-mediated delivery of ITGA9 siRNA downregulated ITGA9 in mammary tumors, significantly reducing angiogenesis, tumor growth, and metastasis [61]. Interestingly, although proteomics confirmed the hydrogel is rich in angiogenic ECM components, we did not detect known classical ITGA9 ligands (e.g., tenascin-C, osteopontin). This suggests a novel, non-classical activation mechanism for ITGA9. Integrin–ligand interactions exhibit redundancy and plasticity, and unidentified ligands within the hydrogel may be involved. Proteomic analysis revealed significant enrichment of Laminin-521 in HCH dECM. The ligand – receptor interactions between laminins and integrins are well established in prior studies. As a key component in basement membrane assembly, Laminin-521 binds to various integrins—including α6β1, α3β1, and α7β1—via its α5 subunit, thereby playing crucial biological roles in processes such as cell migration and tissue repair [62]. Although direct binding between Laminin-521 and integrin α9β1 has not been experimentally confirmed, their biological functions exhibit substantial overlap. Notably, physiological degradation in vivo may generate bioactive short peptides from Laminin-521 subunits, which could subsequently participate in activating ITGA9 and its downstream signaling pathways. Besides biochemical signals, the mechanical properties of the hydrogel may also contribute to ITGA9 activation [63]. Integrins can act as mechanosensors, and our composite hydrogel may provide an ideal microenvironment for ITGA9-mediated mechanotransduction—consistent with our goal of enhancing mechanical properties. Furthermore, elevated ITGA9 expression not only explains the pro-angiogenic mechanism of the HCH gel but may also reflect a process relevant to HCH pathogenesis. HCH ECs may respond to their own ECM signals by highly expressing ITGA9, thereby driving tumor growth. Although hypoxia is often implicated in VEGFA upregulation, RNA sequencing revealed no changes in hypoxia-inducible factors, and no hypoxia-related pathways were enriched—further supporting the central role of the ITGA9–FAK–ERK1/2 axis in HCH gel-induced angiogenesis. In summary, our findings underscore the promise of designing biomaterials that specifically target and upregulate ITGA9 as an innovative direction for next-generation pro-angiogenic therapies.

Despite the promising results, this study has several limitations that warrant consideration and present opportunities for future research. First, while the incorporation of GelMA significantly enhanced the mechanical stability and handling properties of the HCH dECM hydrogel, it concurrently compromised the native thermosensitive gelation and straightforward injectability of the pure dECM component. Second, the remarkable pro-angiogenic effect was attributed to the complex composition of HCH dECM, yet this very complexity poses a challenge in pinpointing the specific ligand(s) responsible for ITGA9 activation. To move from correlation to causation, subsequent studies should employ targeted approaches such as affinity purification-mass spectrometry to identify direct binding partners for ITGA9 within the HCH gel. Additionally, systematic quality control for HCH specimens should be implemented based on key protein or peptide markers to ensure consistent therapeutic efficacy and reliability in future clinical applications.

## 5 Conclusion

In this study, we developed a novel bioactive composite hydrogel scaffold by integrating HCH dECM with GelMA. This hydrogel construct establishes a favorable microenvironment that actively modulates the biological behavior of HUVECs and facilitating their assembly into functional vascular networks. In vivo, the HCH gel potently induced robust capillary formation, offering a rich vascular supply to support tissue regeneration and engineered organoids. Mechanistically, we identified integrin α9β1 as a key transmembrane receptor on ECs that interacts with the HCH gel, and demonstrated that the ITGA9–FAK–ERK1/2 signaling axis plays a central role in driving angiogenesis. These findings position the HCH gel as a highly promising human-derived biomaterial with strong potential for applications in regenerative medicine and tissue engineering.

## Funding

This study was supported by the 10.13039/501100001809National Natural Science Foundation of China (Grant No. 31370989) and the 10.13039/501100012131Department of Science and Technology of Liaoning Province, China (Grant Nos. 2023JH2/20200140 and 2023JH2/20200147).

## CRediT authorship contribution statement

**Zongbo Dai:** Conceptualization, Data curation, Formal analysis, Investigation, Methodology, Visualization, Writing – original draft. **Xuejian Li:** Investigation. **Meiqi Jin:** Investigation. **Wanting Liu:** Investigation. **Xiaohang Li:** Investigation, Resources. **Chengshuo Zhang:** Investigation, Resources. **Feng Li:** Investigation, Resources. **Jianzhen Lin:** Investigation, Resources. **Ziyi Zhou:** Investigation, Resources. **Xiaoting Sun:** Funding acquisition, Project administration, Supervision, Writing – review & editing. **Tianlin Wang:** Funding acquisition, Project administration, Supervision, Writing – review & editing. **Huazhe Yang:** Funding acquisition, Project administration, Supervision, Writing – review & editing. **Jialin Zhang:** Funding acquisition, Project administration, Supervision, Writing – review & editing.

## Declaration of competing interest

The authors declare that they have no known competing financial interests or personal relationships that could have appeared to influence the work reported in this paper.

## Data Availability

Data will be made available on request.
