# Supplementary material for: Hepatic cavernous hemangioma decellularized extracellular matrix/GelMA composite hydrogel promotes angiogenesis via the ITGA9–FAK–ERK1/2 axis
Source: Mater Today Bio. 2026 Feb 28;38:102976. doi: 10.1016/j.mtbio.2026.102976 (PMC13049530; doi:10.1016/j.mtbio.2026.102976)

**Supplemental Table 1** Detailed composition of the composite hydrogels (1 mL).

| Final concentration | 10% (w/v) GelMA | HCH dECM solution |
| --- | --- | --- |
| 5% GelMA with 2.5 mg/mL HCH dECM | 500 μL | 5 mg/mL, 500 μL |
| 5% GelMA with 5 mg/mL HCH dECM | 500 μL | 10 mg/mL, 500 μL |
| 5% GelMA with 7.5 mg/mL HCH dECM | 500 μL | 15 mg/mL, 500 μL |
| 5% GelMA with 10 mg/mL HCH dECM | 500 μL | 20 mg/mL, 500 μL |

**Supplemental Table 2** Primer sequences in real-time quantitative PCR experiments.

| Gene | Primer sequence (5’-3’) | Product length (bp) |
| --- | --- | --- |
| VEGFA (human) | F: CAACAAATGTGAATGCAGACCAA | 157 |
|  | R: GCTCCAGGGCATTAGACAGC |  |
| PDGFB (human) | F: TGAGCAGGAATGGTGAGATGT | 144 |
|  | R: CCCCATCTTCCTCTCCGGG |  |
| TGFB1 (human) | F: GGAAATTGAGGGCTTTCGCC | 90 |
|  | R: CCGGTAGTGAACCCGTTGAT |  |

**Supplemental Table 3** Interfering sequences against ITGA9.

| Name | Interfering seqence (5’-3’) |
| --- | --- |
| siITGA9-1 (human) | Sense: CCCGAUCGUGUUUGAAGCA |
|  | Antisense: UGCUUCAAACACGAUCGGG |
| siITGA9-2 (human) | Sense: CCUAAUCGACUCUCAUCUG |
|  | Antisense: CAGAUGAGAGUCGAUUAGG |
| siITGA9-3 (human) | Sense: GAACGACGAAGUGAUCAUG |
|  | Antisense: CAUGAUCACUUCGUCGUUC |

Supplemental Fig. 1. Characterization of HCH dECM hydrogel and HCH dECM/GelMA composite hydrogel (HCH gel). (A) Pore size and porosity of the 10 mg/mL HCH dECM hydrogel and the HCH gel (composed of 5 mg/mL HCH dECM and 5% GelMA). (B) Volcano plots illustrating the significantly differentially expressed proteins between native HCH and HCH dECM. (C) GO enrichment analysis of significantly enriched signaling pathways. (D) KEGG enrichment analysis. (n = 3, ***p < 0.001, ****p < 0.0001).


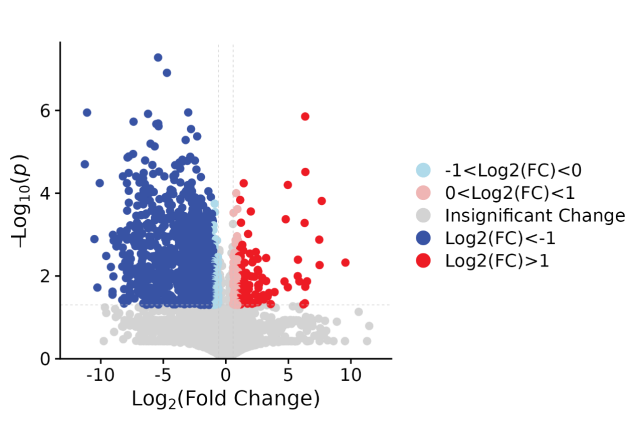


**B**

**C**

**D**


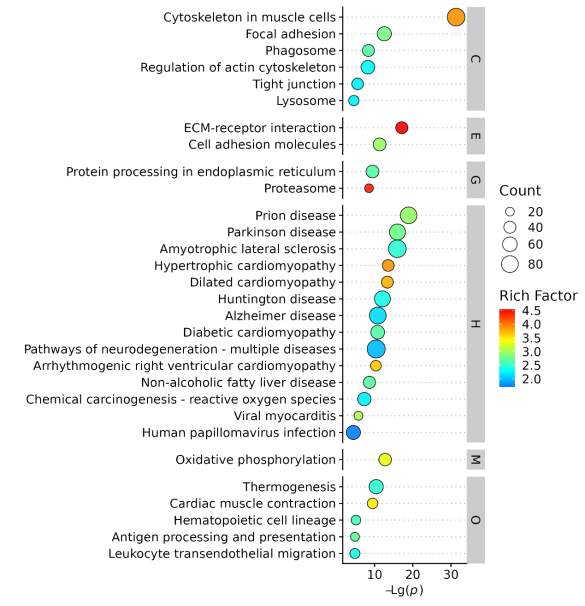

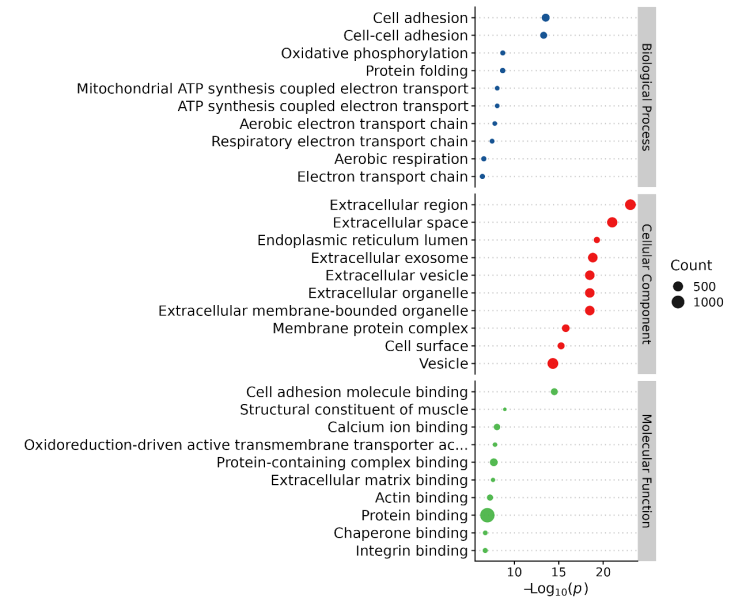


**A**

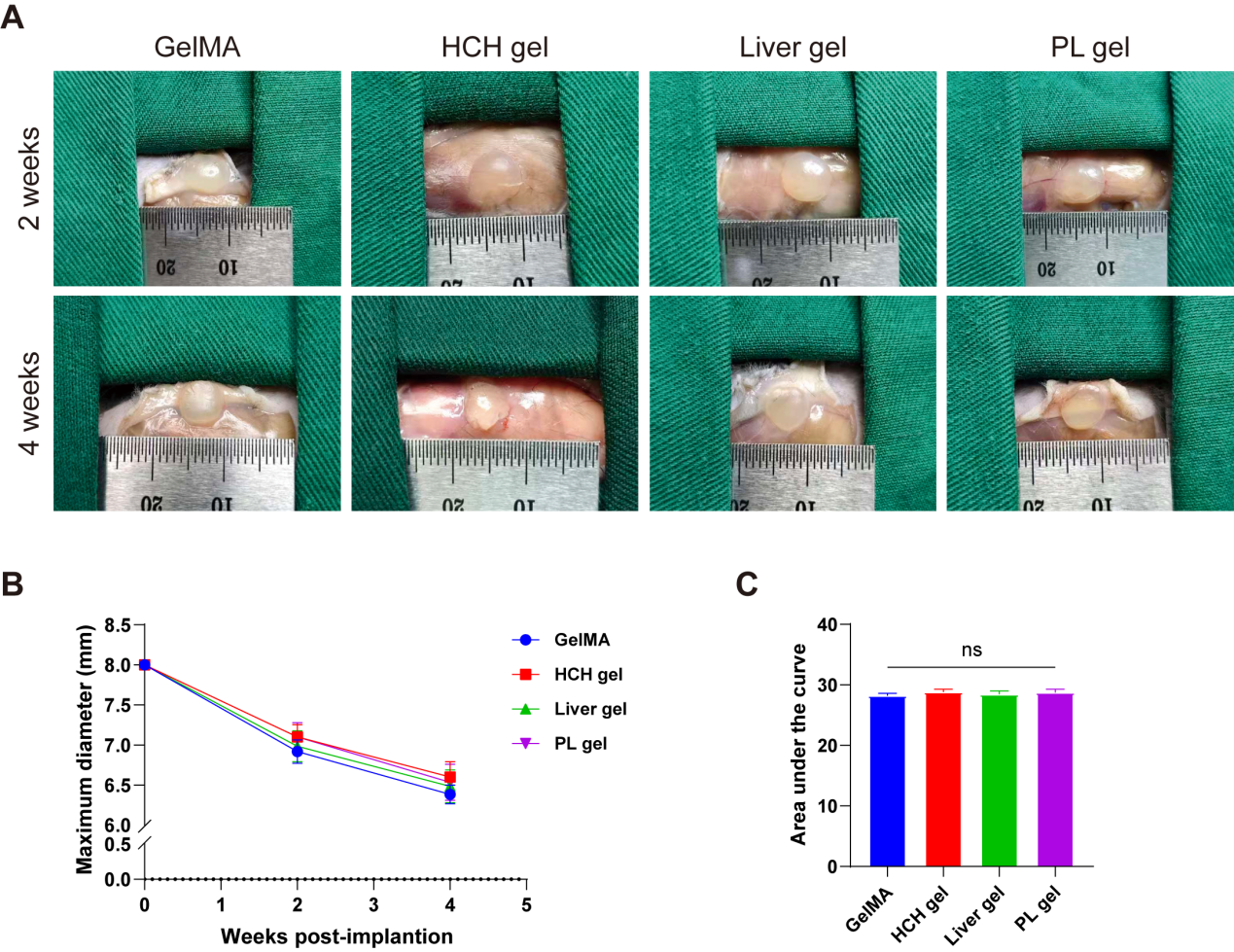


Supplemental Fig. 2. In vivo degradation of hydrogels. (A) Gross specimens of hydrogels harvested from the mouse subcutaneous space at 2 and 4 weeks post-implantation. (B) Degradation kinetics of the hydrogels over time. (C) Quantification of the area under the curve for the degradation profiles from graph B. (n = 6).

Supplemental Fig. 3. Biocompatibility of the HCH gel in vivo. H&E stained images of the heart, liver, spleen, lung, and kidney (scale bar = 100 µm).

**Heart**


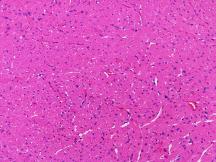

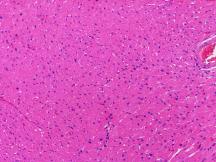

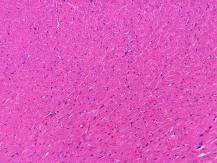

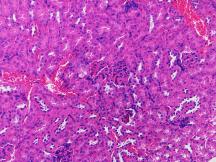

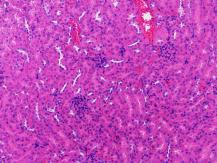

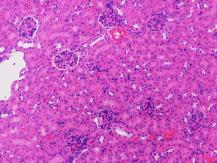

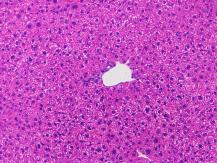

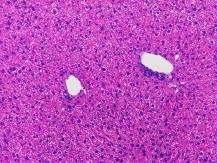

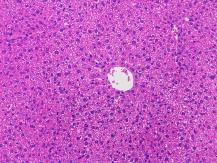

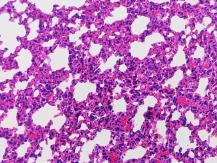

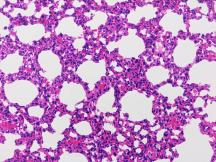

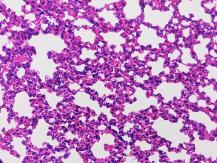

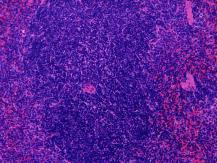

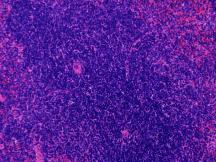

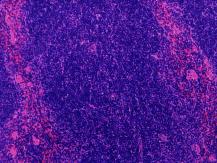


**Liver**

**Spleen**

**Lung**

**Kidney**

**Control**

**GelMA**

**HCH gel**

Supplemental Fig. 4. RNA sequencing analysis of HUVECs. (A) GO enrichment analysis of signaling pathways enriched in HUVECs cultured on HCH gel compared with those on GelMA. (B) KEGG enrichment analysis of the same comparison. (C) Clustered heatmap of the intersection of significantly differentially expressed genes between the HCH gel vs. control and HCH gel vs. GelMA comparisons (n = 3).

**A**

**B**


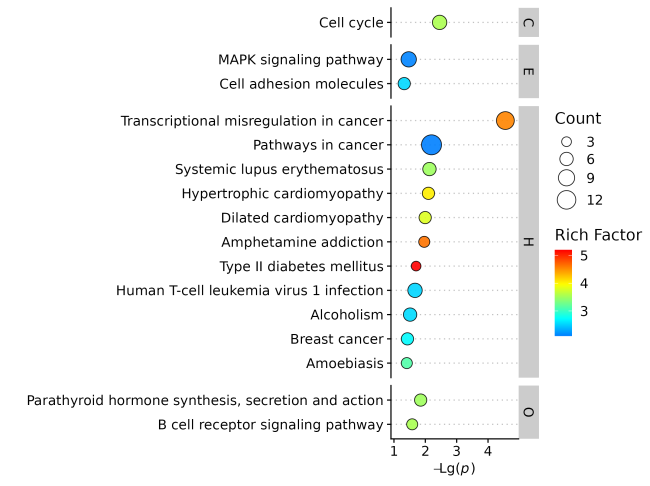

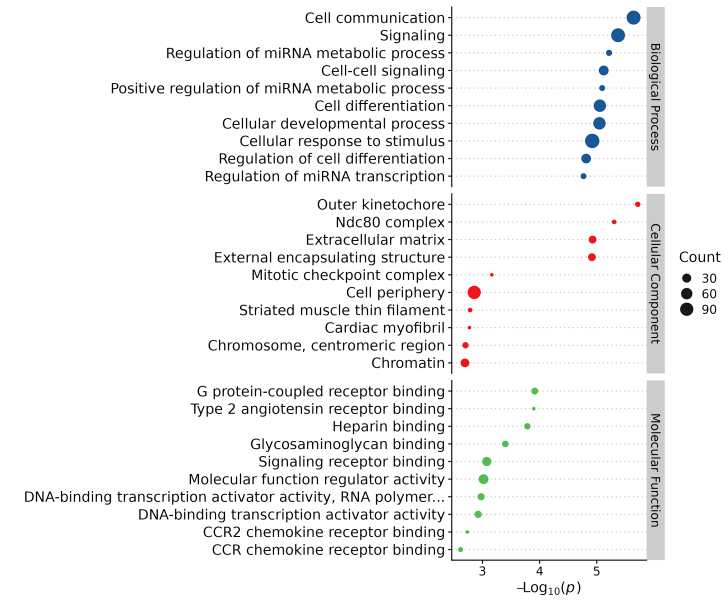

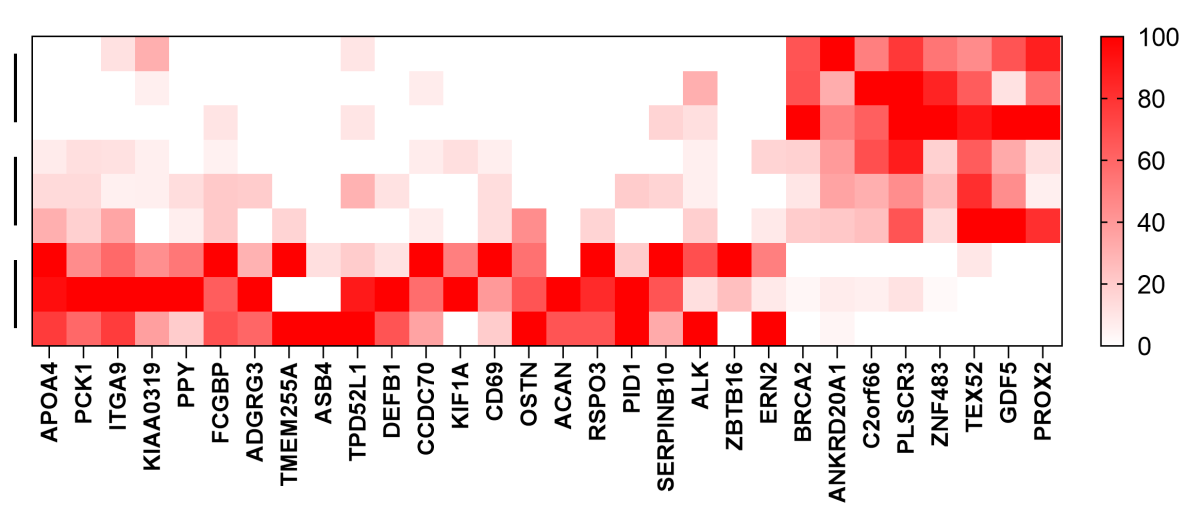


**C**

**Control**

**GelMA**

**HCH gel**

Supplemental Fig. 5. ITGA9 knockdown efficiency in HUVECs by siRNA transfection. (A) qRT-PCR analysis of ITGA9 mRNA expression in HUVECs. (B) Western blot analysis of ITGA9 protein expression. (C) Quantification of Western blot results. (n = 3, *p < 0.05, **p < 0.01, ***p < 0.001, ****p < 0.0001).


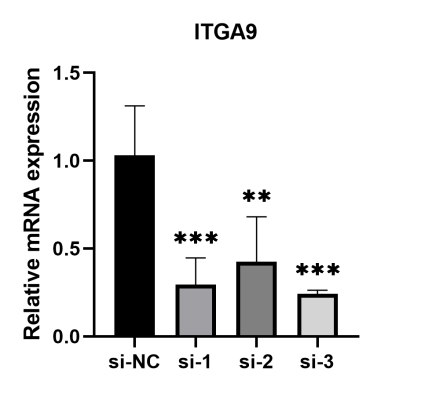


**A**

**B**


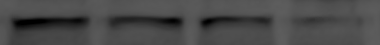


**Si-NC**

**Si-1**

**Si-2**

**Si-3**

**ITGA9**

**β-Tubulin**

**135 KDa**

**50 KDa**


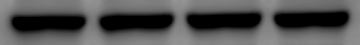


**C**


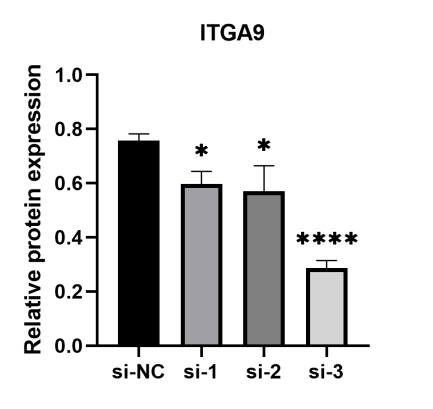

Supplement: Multimedia component 1 [file mmc1.docx]
